# Effects of Decentralized Sequencing on National *Listeria monocytogenes* Genomic Surveillance, Australia, 2016–2023

**DOI:** 10.3201/eid3113.241357

**Published:** 2025-05

**Authors:** Patiyan Andersson, Sally Dougall, Karolina Mercoulia, Kristy A. Horan, Torsten Seemann, Jake A. Lacey, Tuyet Hoang, Lex E.X. Leong, David Speers, Louise Cooley, Karina Kennedy, Rob Baird, Rikki Graham, Qinning Wang, Avram Levy, Dimitrios Menouhos, Norelle L. Sherry, Susan A. Ballard, Vitali Sintchenko, Amy V. Jennison, Benjamin P. Howden

**Affiliations:** The University of Melbourne, Melbourne, Victoria, Australia (P. Andersson, S. Dougall, K. Mercoulia, K.A. Horan, T. Seeman, J.A. Lacey, T. Hoang, N.L. Sherry, S.A. Ballard, B.P. Howden); SA Pathology, Adelaide, South Australia, Australia (L.E.X. Leong); Queen Elizabeth II Medical Centre, Perth, Western Australia, Australia (D. Speers, A. Levy); Royal Hobart Hospital, Hobart, Tasmania, Australia (L. Cooley); Canberra Health Services, Australian National University Medical School, Canberra, Australian Capital Territory, Australia (K. Kennedy); Territory Pathology, Royal Darwin Hospital, Darwin, Northern Territory, Australia (R. Baird, D. Menouhos); Queensland Public Health and Scientific Services, Queensland Health, Brisbane, Queensland, Australia (R. Graham, A.V. Jennison); Institute of Clinical Pathology and Medical Research, NSW Health Pathology, Sydney, New South Wales, Australia (Q. Wang, V. Sintchenko); Austin Health, Heidelberg, Victoria, Australia (N.L. Sherry, B.P. Howden); Sydney Institute for Infectious Diseases, The University of Sydney, Sydney (V. Sintchenko)

**Keywords:** Listeriosis, bacteria, genomic surveillance, enteric infections, food safety, public health surveillance, pathogen genomics, listeria monocytogenes, Australia

## Abstract

We assessed turnaround times in the national *Listeria monocytogenes* genomic surveillance system in Australia before and after decentralized sequencing. Using 1,204 samples collected during 2016–2023, we observed statistically significant reductions in median time from sample collection to issuance of national genomic surveillance report to 26 days, despite sample numbers doubling in 2022 and 2023. During 2016–2018, all jurisdictions referred samples to the National Listeria Reference Laboratory for sequencing and analysis, but as jurisdictional sequencing capacity increased, 4 jurisdictions transitioned to sequencing their own samples and referring sequence data to the national laboratory. One jurisdiction had well-established genomics capacity, transitioned without noticeable disruption, and continued to improve. Another 3 jurisdictions initially had increased turnaround times, highlighting the need for defined sequence referral mechanisms. Overall, timeliness and throughput improved, and sequencing decentralization strengthened Australia’s genomic surveillance system while maintaining timeliness. The practices described could be beneficial and achievable in other countries.

*Listeria monocytogenes* is an etiologic agent for gastroenteritis but can also cause serious invasive disease (*1*). The incidence of invasive listeriosis is relatively low, but the case-fatality rate is one of the highest among foodborne infections (*2*,*3*). The severity of *L. monocytogenes* infection, along with its ubiquitous environmental presence and frequent outbreaks from commercially manufactured foods, results in major social and economic impacts (*4*–*6*). Collecting detailed information for both the case and the pathogen enhances the success of public health investigations.

Whole-genome sequencing (WGS) provides high-resolution characterization of pathogens and has been shown to be critical in identifying outbreak clusters, separating outbreaks from endemic cases, and linking food and environmental samples to human cases with greater confidence (*7*,*8*). Consequently, WGS has been implemented for routine surveillance of *L. monocytogenes* in several countries, including Australia (*9*–*16*).

In Australia, invasive listeriosis has been a notifiable disease since 1991 and is recorded in the National Notifiable Diseases Surveillance System (NNDSS) (*17*). Public health monitoring and action is managed by OzFoodNet, the national foodborne disease surveillance network. In 2010, the National Enhanced Listeriosis Surveillance System (NELSS) was established to collate both enhanced epidemiologic data from cases and molecular laboratory data from isolates (*18*,*19*). Once NELSS was established, the National Listeria Reference Laboratory (NLRL), based at the Microbiological Diagnostic Unit Public Health Laboratory (MDU PHL) in the state of Victoria, was tasked with providing national molecular characterization of all referred *L. monocytogenes* samples, including typing with pulsed-field gel electrophoresis (PFGE). In July 2015, the NLRL commenced routine WGS for all referred samples and, after a 12-month trial of parallel use with PFGE, WGS became the preferred typing method (*20*). The NLRL also conducts centralized genomic analysis and issues a national genomic surveillance report.

As a federation, Australia’s 8 jurisdictions are independently responsible for their public health activities, including pathogen genomics. Since 2016, genomic sequencing capacity has expanded in Australia; MDU PHL continued WGS for Victoria, and 4 additional jurisdictions successively became responsible for their own *L. monocytogenes* WGS during 2018–2023. The other 3 jurisdictions still refer samples to the NLRL for WGS. We investigated the timeliness and continued evolution of national *L. monocytogenes* surveillance from the perspective of the transition to a decentralized sequencing model.

## Materials and Methods

### Setting

Australia is a federated nation composed of 8 jurisdictions and had a combined estimated residential population of 27,000,000 in 2023 (*21*). We obtained annual listeriosis incidence rates from the NNDSS dashboard (*17*).

**Figure 1:**
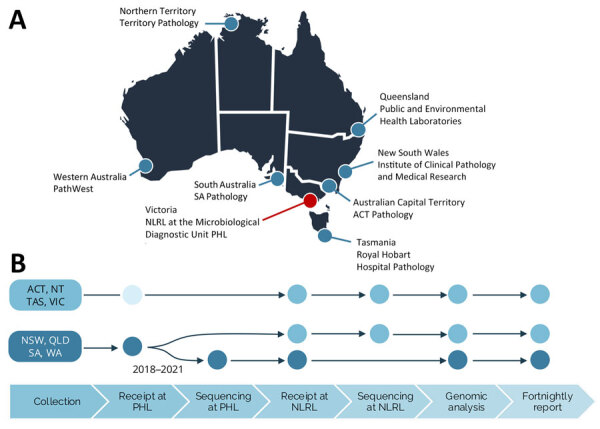
National decentralized sequencing system for *Listeria monocytogenes* genomic surveillance, Australia. A) Eight jurisdictional public health laboratories contributing to genomic surveillance. The NLRL is based at the Microbiological Diagnostic Unit (MDU) PHL in the state of Victoria. B) Overview of the steps in the national genomic surveillance system; dots indicate where sample processing occurs. The process is the same for human and nonhuman samples. For jurisdictions ACT, NT, TAS, and VIC, sequencing is performed by the NLRL at the Microbiological Diagnostic Unit PHL. The unfilled circle indicates that some samples are referred directly from the primary pathology laboratory to NLRL. For jurisdictions NSW, QLD, SA, and WA, the referral pathway transitioned during 2018–2021 from sequencing performed by the NLRL to jurisdictional sequencing and referral of sequences for genomic analysis. ACT, Australian Capital Territory; NLRL, National Listeria Reference Laboratory; NSW, New South Wales; NT, Northern Territory; PHL, public health laboratory; QLD, Queensland; SA, South Australia; TAS, Tasmania; VIC, Victoria; WA, Western Australia.

In Australia, samples from listeriosis notifications and relevant positive food and environmental samples are forwarded to public health laboratories (PHL) in each jurisdiction for confirmation, and PHLs subsequently refer sequences or isolates to the NLRL for national genomic analysis (Figure 1). Sequencing and bioinformatic analysis of *L. monocytogenes* at the NLRL are to ISO 17025 and ISO 15189 standards and accredited by the Australian National Association of Testing Authorities (https://www.nata.com.au).

### Study Sample Dataset

This study included all *L. monocytogenes* samples referred to the NLRL for sequencing and all *L. monocytogenes* sequences referred by PHLs during 2016–2023, representing the first complete year of WGS to the most recent full year of data. Sample metadata included referring laboratory, residential jurisdiction of the case, and sample source categorized as human, food, or environmental. All sequence data were generated on Illumina (https://www.illumina.com) platforms, and the NLRL analysis workflow applied quality control thresholds of >40× coverage, *L. monocytogenes* species detected, and genome size within 10% of expected maximum genome size. We assessed timeliness of the genomic surveillance system by using temporal data, including date sample was collected, date sample was received at the jurisdictional PHL, date sample was sequenced, date NLRL received sample or sequence data, and date NLRL issued national genomics report.

### Statistical Analysis

We used a Shapiro-Wilk test to assess for normality in the processing times at each stage, for each year, and for each jurisdiction. We excluded years with <3 observations from the normality testing. Because most of the dataset was not normally distributed, we used a nonparametric Kruskal-Wallis test to assess differences in processing times across years and, where statistically significant (p<0.05), performed a Dunn’s posthoc test with Bonferroni correction on the pairwise comparison of years for each jurisdiction.

## Results

### Notifications and Study Sample Set

Listeriosis became a notifiable disease in Australia in 1991. The average number of notifications recorded in the NNDSS was 65.2 (range 35–93) cases/year. The annual incidence rate of listeriosis remained relatively stable since 1991, ranging from 0.2 to 0.4/100,000 population.

During 2016–2023, Australia had 545 notified listeriosis cases, and yearly case numbers ranged from 43 to 89. We included a total of 543 sequences from 508 individual cases in the study, representing sequences from an average of 93.2% (range 87.3%–100%) of cases per year. We also included an additional 418 sequences of *L. monocytogenes* cultured from food samples and 243 sequences from environmental samples, bringing the complete dataset to 1,204 samples.

**Figure 2:**
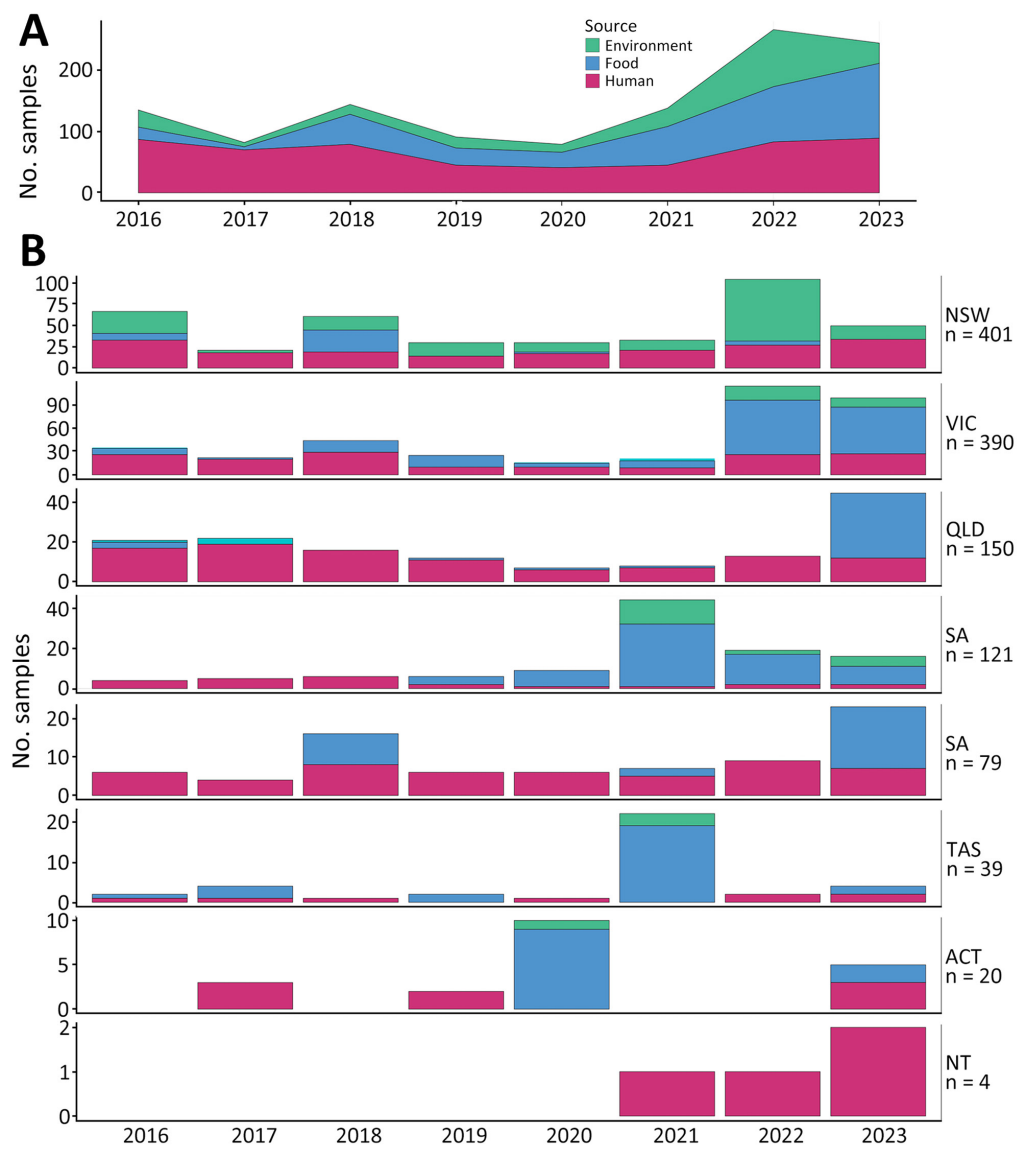
Summary of *Listeria* *monocytogenes* samples included in a study of effects of decentralized sequencing on national *L. monocytogenes* genomic surveillance, Australia, 2016–2023. A) Number of samples per year by source; B) number of samples per jurisdiction per year and source. Total number of samples per jurisdiction are provided; note varying scales of the y-axes. A notable increase in samples from food and environmental sources has occurred since 2021. ACT, Australian Capital Territory; NSW, New South Wales; NT, Northern Territory; QLD, Queensland; SA, South Australia; TAS, Tasmania; VIC, Victoria; WA, Western Australia.

Samples from the 2 most populous jurisdictions, New South Wales (NSW) and Victoria (VIC), made up 65% of the dataset, and Queensland (QLD) and South Australia (SA) comprised another 22.5% (Figure 2). All jurisdictions submitted isolates or sequences from human, food, and environmental sources, except Northern Territory (NT) and Western Australia (WA). However, the distribution was uneven; VIC had a high (49%) percentage of food samples and NSW had a high (43%) percentage of environmental samples, demonstrating some differences in investigation practices. We noted a marked increase in the number of submissions from 2021 onward, mainly driven by increases from food and environmental sources.

### Sample Referral Pathways

Samples from notified cases and food and environmental sources from all jurisdictions are referred to the NLRL for inclusion in genomic analysis (Figure 1, panel B). Before 2018, all jurisdictions referred either primary samples (food or environmental) or cultured isolates to the NLRL where, after *L. monocytogenes* culture, if required, isolates were subject to WGS and bioinformatic analysis. PHLs gradually transitioned to performing sequencing locally and referring *L. monocytogenes* genome sequences to the NLRL for inclusion in the national analysis. By 2023, four jurisdictions had transitioned to local sequencing: QLD in 2018, NSW in 2019, SA in 2020, and WA in 2021. The NLRL processed samples from VIC and continued to support NT, Australian Capital Territory (ACT), and Tasmania (TAS) with WGS services.

### Genomic Surveillance Reporting

The genomic surveillance report includes samples collected within a 24-month rolling window and provides phylogenetic and clustering data, including historical data for context when relevant. Single-linkage clustering is performed and reported as highly related if the pairwise difference is <5 single-nucleotide polymorphisms, and possibly related if the pairwise difference is 6–20 single-nucleotide polymorphisms. Since genomic surveillance began in 2015, NLRL has issued >200 formal national *L. monocytogenes* genomic surveillance reports. Those reports are distributed to the referring PHLs, and to the national coordinating OzFoodNet epidemiologists and jurisdictional OzFoodNet epidemiologists. Reports are issued every 2 weeks, but genomic analysis is conducted weekly at a minimum and more frequently during outbreak investigations (Appendix 1). Critical findings from analyses are communicated immediately to epidemiologists via phone or email.

### National Genomic Surveillance System Timeliness

#### Overall System Timeliness

**Figure 3:**
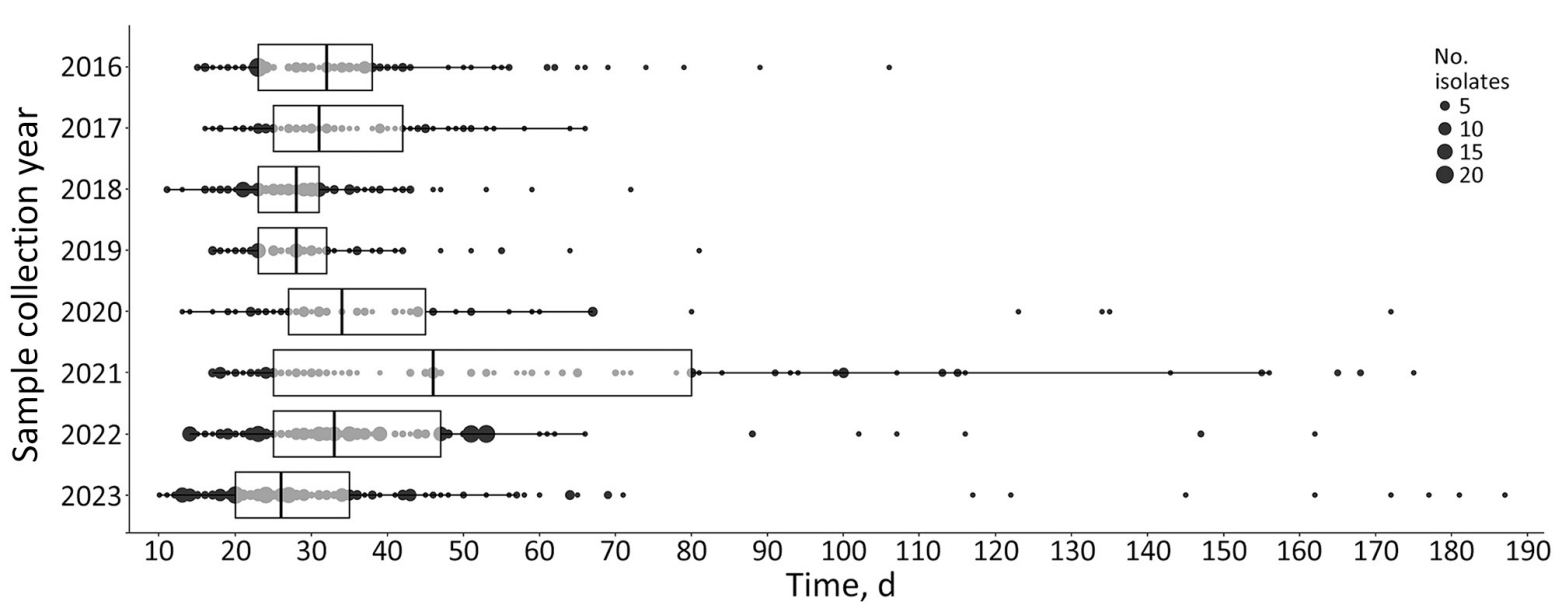
Box and whisker representation of end-to-end timeliness in a study of effects of decentralized sequencing on national *Listeria monocytogenes* genomic surveillance, Australia, 2016–2023. Time represents date of sample collection to date genomic surveillance report was issued, by year. Boxplots show medians (vertical lines within boxes), first and third quartiles (box left and right edges), and 1.5× interquartile range from each quartile (whiskers); points outside that range are considered outliers. Underlying data are shown as dots, and dot size corresponds to the number of samples at each timepoint. Statistically significant differences (p<0.001) between the years were calculated by using Kruskal-Wallis χ^2^ test. Dunn’s posthoc test showed statistically significant (p<0.001) increases in times for years 2020 and 2021, compared with previous years (p = 0.029), and subsequent significant decreases in times in 2022 and 2023 (adjusted p<0.001). Median time in 2023 was 26 days, compared with a median of 32 days in 2016.

In the dataset of 1,204 samples, we excluded 39 historical samples because those samples were not sequenced in real time. Thus, the timeliness analysis included 1,165 samples. We calculated the end-to-end turnaround times of the surveillance system, from date of sample collection to issuance of the national genomic surveillance report (Figure 3). We observed a pattern of pre–COVID-19 pandemic improvements but a statistically significant increase in turnaround times in 2020 and 2021 compared with previous years (pairwise comparisons years 2016 to 2021, adjusted p<0.001 to p = 0.029), and subsequent time reductions in 2022 and 2023 (adjusted p<0.001). We also observed a statistically significant improvement in timeliness between 2016 (median 32 days) and 2023 (median 26 days) (adjusted p<0.001).

We noted differing patterns of timeliness across the jurisdictions (Appendix 2 Figure 1), and all jurisdictions, except NT, showed statistically significant changes over the years (p<0.001–0.007). NT had too few observations for analysis. VIC was the most stable, consistently maintaining median turnaround times of 24–27 days, although that range increased in the 2 most recent years, and VIC had a significantly higher median in 2022 (p = 0.032) compared with other all years (p<0.001). Of the jurisdictions that have transitioned from referring samples to referring sequences, NSW remained consistent over time but had larger variations in median turnaround times, 24–42 days, and higher upper limits, 60–70 days, not considering outliers (Figure 3). NSW and QLD demonstrated statistically significant improvements in 2023 compared with 2016 (Table). However, QLD, SA, and WA all showed increases in median and range of turnaround times immediately after transitioning to referring sequence data instead of isolates.

**Table:** Sample processing times used in a study of effects of decentralized sequencing on national *Listeria monocytogenes* genomic surveillance, Australia, 2016–2023*

Collection year	Median time, d (range)							
	ACT	NSW	NT	QLD	SA	TAS	VIC	WA
Overall referral time†								
All sample sources								
2016	ND	29 (15–106)	ND	36 (30–55)	37 (33–61)	34 (29–39)	25 (15–37)	44 (36–65)
2023	36 (36–43)	24 (10–65)	75 (28–122)	20 (12–43)	45 (14–71)	30 (18–32)	26 (12–56)	50 (26–187)
p value	ND	0.003	ND	<0.001	NS	NS	NS	NS
Primary referral time‡								
Human samples								
2016	ND	5 (1–14)	ND	4 (3–7)	2 (2–3)	4 (4)	5 (2–10)	6 (2–10)
2023	13 (12–13)	6 (0–20)	3 (3)	5 (2–6)	3 (1–5)	9 (8–9)	3 (1–7)	4 (0–10)
p value	ND	NS	ND	NS	NS	NS	<0.001	NS
Food samples								
2016	ND	1 (1)	ND	1 (0–11)	ND	19 (19)	4 (0–14)	ND
2023	15 (15)	ND	ND	0 (0–2)	9 (6–17)	9 (9)	4 (0–25)	32 (0–129)
p value	NS	ND	ND	NS	ND	0.026	NS	ND
Environmental samples								
2016	ND	1 (0–2)	ND	1 (1)	ND	ND	0	ND
2023	ND	1 (1)	ND	ND	29 (29)	ND	1 (0–6)	ND
p value	ND	NS	ND	ND	ND	ND	NS	ND
Sequencing time§								
Human samples								
2016	ND	10 (3–18)	ND	10 (8–14)	10 (5–13)	11 (11)	10 (6–18)	12 (10–20)
2023	13	5 (1–12)	11 (10–11)	8 (2–12)	3 (2–5)	8 (6–11)	8 (5–11)	6 (1–12)
p value	ND	<0.001	ND	0.014	NS	NS	0.003	NS
Food samples								
2016	ND	3 (3–10)	ND	14 (10–18)	ND	16 (16)	20 (15–20)	ND
2023	9 (9)	15 (5–15)	ND	7 (6–14)	9 (6–14)	8 (7–9)	11 (6–25)	7 (2–8)
p value	ND	<0.001	ND	NS	ND	NS	0.002	ND
Environmental samples								
2016	ND	10 (3–14)	ND	14 (14)	ND	ND	17 (17)	ND
2023	ND	5 (2–5)	ND	ND	6 (6)	ND	16 (9–21)	ND
p value	ND	<0.001	ND	ND	ND	ND	NS	ND
Sequence referral time§								
All sample sources								
2016	ND	20 (9–96)	ND	27 (13–41)	25 (17–52)	ND	ND	34 (21–48)
2023	ND	12 (4–54)	ND	12 (3–19)	26 (7–31)	ND	ND	12 (7–26)
p value	ND	<0.001	ND	<0.001	NS	ND	ND	0.007
Genomic analysis time^#^								
2016	ND	11 (0–14)	ND	8 (1–11)	10 (4–17)	9 (4–14)	8 (3–17)	9 (2–11)
2023	19 (15–19)	8 (1–13)	7 (2–12)	1 (1–19)	9 (3–14)	12 (4–15)	9 (1–21)	14 (3–18)
p value	ND	0.025	ND	0.002	NS	NS	NS	<0.001

*Times are shown as median (range) in days for each jurisdiction for years 2016 and 2023, and adjusted p values from Dunn’s post-hoc tests of pairwise comparisons. Range defined as data points within 1.5 from each quartile, with points outside interquartile range considered outliers. ACT, Australian Capital Territory; ND, no data available; NS, not statistically significant; NSW, New South Wales; NT, Northern Territory; QLD, Queensland; SA, South Australia; TAS, Tasmania; VIC, Victoria; WA, Western Australia. †Date of collection to date genomic surveillance report issued. ‡Date of collection to date sample received at jurisdictional public health laboratory. §Date received at jurisdictional public health laboratory to date sequenced. ¶Date received at jurisdictional PHL to date sequence available for bioinformatic analysis at the National Listeria Reference Laboratory. #Date received at national *Listeria* reference laboratory to date national genomic report issued.

When comparing the 2016 and 2023 median turnaround times for only human samples, we noted most jurisdictions improved timeliness (Appendix 2 Figure 2). We noted statistically significant variations only in NSW (p<0.001) and WA (p<0.02), despite the appearance of large variations in the SA and QLD data for human sequences.

We also assessed time before and after transitioning to sequence referrals for the 4 relevant states (Appendix 2 Figure 3). Although NSW did not show any difference before and after transition, the 3 other states had significant differences in median turnaround times (p<0.001). Only the 2 earliest transitioning states, QLD (adjusted p<0.001) and NSW (adjusted p = 0.003), achieved medians in 2023 that were significantly lower than those of 2016.

#### Primary Referral Time

The time for collection of human samples to referral of samples to the jurisdictional PHLs was consistent over time; median times were ≈5 days and upper limits <10 days for most jurisdictions except NSW (Table; Appendix 2 Figure 4). In VIC, SA, TAS, and particularly WA, referral of food samples had higher upper time limits. However, VIC had a reduced median because a large number were referred in <1 day. We also observed that trend in NSW and QLD.

#### Sequencing Times

Median processing times, from sample receipt at the PHL to date sequencing preformed, were relatively consistent (10 days) across jurisdictions, with some minor improvements between 2016 and 2023 (Table; Appendix 2 Figure 5). The sequence processing times include potential culturing of samples received as primary specimen. Processing times for human samples improved considerably between 2016 and 2023 in NSW, SA, VIC, and WA. The sequence processing times for food samples were similar to those of human samples in all jurisdictions except VIC. The difference for VIC can be explained by the referral workflow, in which the NLRL in VIC would predominantly receive cultured food isolates from other jurisdictions for sequencing, but NLRL received local VIC samples as primary specimens that require culture and isolation before sequencing. The considerable improvements we noted in VIC for the 2 most recent years can partially be attributed to a larger number of local food samples received as cultured isolates.

#### Effects of Transition to Sequence Referral

To compare the effect of transitioning to sequence referral, we considered the entire process from sample collection to bioinformatic analysis, thereby accounting for processing times at each phase, including culturing and isolation, sequencing, and sample or sequence referral, either at the jurisdictional PHL or the NLRL (Appendix 2 Figure 6). Median processing time at each of the 4 jurisdictions before and after transition showed significant reductions associated with referral of sequences for NSW (adjusted p<0.001) and QLD (adjusted p<0.001), but a significant increase in processing times for referred sequences in SA (adjusted p = 0.015). We found no overall significant differences for WA. We noted considerable variation in processing times between years for QLD, SA, and WA, but NSW was more stable. Comparing the extreme timepoints of 2016 and 2023, we observed statistically significant reductions for NSW (adjusted p<0.001), QLD (adjusted p<0.001), and WA (adjusted p = 0.007).

#### Genomic Analysis Times

We calculated the genomic analysis times on the basis of the date a sequence was available (either sequencing completed at the NLRL or PHL sequence received by the NLRL) and the date the fortnightly genomic surveillance report was issued (Appendix 2 Figure 7). Thus, times varied depending on when in the reporting cycle the sequence became available. Genomic analysis and reporting were consistent and timely across the study period; 84.4% (983/1,165) of samples were reported within a 14-day reporting cycle and 99.4% (1,158/1,165) of samples reported within 2 reporting cycles. 

Given the potential effect of the fortnightly reporting cycle, we analyzed the time from sample collection to a sequence being available for analysis and reporting. The pattern for all jurisdictions remained unchanged, but the shortest times in 2023 were just 5–8 days for NSW, QLD, VIC, and SA (data not shown).

## Discussion

We describe the maturation of a multijurisdictional genomic surveillance system for *L. monocytogenes* in Australia and the effects of transitions to decentralized sequencing of isolates as local genomic capacity improved. The overall median time for genomic data to be available was 32 days in 2016, but 2023, the last year in the review, demonstrated the lowest recorded median at 26 days. That difference is a marked improvement when compared with the predecessor analysis method of PFGE, in which the median time from notification to data availability to NELSS during 2010–2013 was 50 days (*18*). We believe the reductions we report are associated with use and increased capacity of automated robotics workflows for WGS; accelerated establishment of strong WGS capacity during the COVID-19 pandemic; and replacement of a physical sample transport step, and potential batching of samples for courier transport, with electronic data transfer. Compared with the first full year of genomic data from the system in 2016, the overall median end-to-end processing time was lower in 2022 and 2023 despite a substantial increase in the number of samples, mainly food and environmental samples. The shift to decentralized sequencing in some jurisdictions might contribute to the ability of the system to manage increased sample volumes without detrimental effects on the timeliness.

Of the 4 jurisdictions that transitioned to sequence referral to NLRL, 3 had considerable increases in overall turnaround times after shifting to PHL sequencing for *L. monocytogenes* but then resumed a downward trajectory in turnaround times. Delays associated with sample batching resulting from limited throughput could be expected during the early stages of establishing sequencing capacity but were not evident from the sequencing times we observed. Instead, we mainly observed delays in referral of sequences to the NLRL. In part, those transitions coincided with the COVID-19 pandemic, during which all PHLs were managing an unprecedented additional workload from real-time SARS-CoV-2 sequencing. Delays might also have been associated with a lack of a national protocol for inclusion of nonhuman samples in the national genomic analyses and variability in the software solutions and processes for sequence referrals. Those observations highlight the need to define and adequately resource sequence referral mechanisms during implementation of local sequencing to ensure optimal turnaround times.

Sequencing capacity was strengthened across all jurisdictions in Australia during the COVID-19 pandemic. After the initial years of the pandemic, sequencing priorities were realigned, which enabled PHLs to apply the enhanced WGS capacity to other pathogens. The capacity for continued improvement speaks well for the future national capability of managing increased volumes of nonhuman samples to improve source identification for *L. monocytogenes*. The benefits of integration of cross-sectoral samples (food and environment) were immediately apparent after the implementation of genomic analysis in Australia, and the sequence from an unresolved case was linked to stone fruits imported from the United States when the sequence was deposited in GenomeTrakr (*22*). By late 2024, Australia had contributed 770 sequences to GenomeTrakr, and that network and the Pathogen Detection Portal continue to be a highly valuable resources for monitoring potential common outbreak sources with international data (*9*,*16*).

The fact that listeriosis notification rates remained stable in Australia over the study period is an indication that public health management of listeriosis remains complex. Although WGS has greatly enhanced the capacity to detect and characterize outbreaks, its effectiveness in reducing overall case numbers is contingent on rapid and comprehensive public health actions, effective control of persistent contamination sources, and improvements in food safety protocols and compliance. Large-scale analysis of international data has shown numerous multinational clusters and emphasized the power of genomics to manage the challenges of persistent environmental contamination and highly interconnected food supply networks (*15*,*23*). The observation of long-term clusters and limited initial epidemiologic signals is further echoed in descriptions from other national genomic surveillance programs (*10*,*11*,*13*,*14*,*24*–*27*). Those findings make a strong argument for coordinated monitoring of *L. monocytogenes* at the global level through consistent and timely data sharing from national surveillance efforts.

Here, we have shown the evolution of timeliness in a longstanding national genomic surveillance system for *L. monocytogenes* and an immediate 30% reduction in median processing time compared with PFGE, then a further 20% reduction to 26 days from sample to notification report observed in 2023. We also demonstrated that surveillance processes can be disrupted and result in delays in data availability during the establishment of decentralized sequencing processes but that those disruptions can be resolved as the capacity matures. Of note, we found that when genomic capacity was already strong in the referring jurisdiction, the transition was managed without noticeable detrimental effects, even in the extraordinary circumstances of the COVID-19 pandemic response. That finding should be considered when making process changes for pathogens that have time-sensitive surveillance objectives. 

In summary, we report the overall picture for decentralized sequencing of *L. monocytogenes* in Australia as one of reduced turnaround times and continued improvement. Decentralization of sequencing strengthened the genomic surveillance system in the country through increased throughput while maintaining timeliness. Such practices could be beneficial and achievable in other countries with sequencing capacity.

Appendix 1 Example national *Listeria monocytogenes* genomic surveillance report for Australia.

Appendix 2 Additional information on effects of decentralized sequencing on national *Listeria monocytogenes* genomic surveillance, Australia, 2016–2023.
