# Supplementary material for: Effects of Decentralized Sequencing on National Listeria monocytogenes Genomic Surveillance, Australia, 2016–2023
Source: Emerg Infect Dis. 2025 May;31(Suppl 1):S89–97. doi: 10.3201/eid3113.241357 (PMC12078541; doi:10.3201/eid3113.241357)
Supplement: Appendix 1 — Example national Listeria monocytogenes genomic surveillance report for Australia. [file 24-1357-Techapp-s1.pdf]

Article DOI: https://doi.org/10.3201/eid3113.241357

EID cannot ensure accessibility for supplementary materials supplied by authors. Readers who have difficulty accessing supplementary content should contact the authors for assistance.

Appendix 1

National Listeria Reference Laboratory

Listeria monocytogenes Surveillance REPORT

REPORTING PERIOD TO: 01/01/2025

To:	Epidemiologists	Report ID:	R2025-123456
		Issue Date:	01/01/2025
Copy to:	Contributing Laboratories	Prepared By:	Epidemiology<br>Bioinformatics
		Authorised By:	Director

1. ANALYSIS SUMMARY

A total of 83 isolates; 35 human cases and 48 non-human sample isolates were received in the 24-week reporting period ending 01/01/2025. All isolates where whole genome sequencing data is available were included in the phylogenetic analysis to provide a more comprehensive representation.

- The phylogenetic tree of all isolates is shown in Section 2. MLST is annotated on the tip labels.
- There are 4 new isolates available for this report.
- There is 1 new recommendation for this reporting period.

Table 1. New isolates added in this report.

Isolate	Primary Lab ID	State	NELSS ID	Source	MLST
2025-xxxxx1		STATE 1	Non-human	Food	1
2025-xxxxx2		STATE 1	Non-human	Food	1
2025-xxxxx3		STATE 3		Human	2
2025-xxxxx4		STATE 4		Human	3

This report is intended solely for the named addressee, is confidential and may contain legally privileged information. The copying or distribution of this document or any information it contains, by anyone other than the addressee, is prohibited.

1.1 New Recommendations:

Epidemiological investigation is HIGHLY recommended for:

- **ST1**
  - Samples 2025-xxxxx1 (STATE 1, NELSS ID: Non-human) and 2025-xxxxx2 (STATE 1, NELSS ID: Non-human) are **highly related to each other** and to case samples 2024-xxxx44 (STATE 1, NELSS ID: xxx) and 2024-xxxx45 (STATE 1, NELSS ID: xxx).

There are no new recommendations for:

- **ST2**
  - Case 2025-xxxxx3 (STATE 3, NELSS ID: xxx) is **not likely related** to other isolates.
- **ST3**
  - Case 2025-xxxxx4 (STATE 4, NELSS ID: xxx) is **not likely related** to other isolates.

This report is intended solely for the named addressee, is confidential and may contain legally privileged information. The copying or distribution of this document or any information it contains, by anyone other than the addressee, is prohibited.

1.2 Sample Submission Details

Table 2. All isolates of *Listeria* from HUMAN sources received in the past 24 weeks for the reporting period ending 01/01/2025.

MDU ID	State	Date Collected	NELSS ID	Molecular Serotype	MLST
	STATE 3	2024-12-31		4b, 4d, 4e	2
	STATE 1	2024-12-27		4b, 4d, 4e	1
	STATE 1	2024-12-27		4b, 4d, 4e	1
	STATE 4	2024-12-27		1/2b, 3b, 7	3
	STATE 1	2024-12-17		1/2a, 3a	37
	STATE 1	2024-12-16		1/2b, 3b, 7	3
	STATE 5	2024-12-15		1/2b, 3b, 7	87
	STATE 2	2024-12-15		1/2b, 3b, 7	3
	STATE 1	2024-12-13		4b, 4d, 4e	1
	STATE 1	2024-12-13		4b, 4d, 4e	1
	STATE 1	2024-12-11		Nontypeable	299
	STATE 2	2024-12-09		Nontypeable	299
	STATE 5	2024-11-28		4b, 4d, 4e	2
	STATE 6	2024-11-26		1/2a, 3a	7
	STATE 2	2024-11-22		1/2a, 3a	321
	STATE 1	2024-11-20		1/2b, 3b, 7	3
	STATE 2	2024-11-20		1/2a, 3a	155
	STATE 2	2024-11-18		1/2b, 3b, 7	87
	STATE 4	2024-11-18		1/2a, 3a	204
	STATE 3	2024-11-10		1/2a, 3a	26
	STATE 1	2024-11-10		1/2a, 3a	7
	STATE 2	2024-11-09		4b, 4d, 4e	1
	STATE 1	2024-10-28		4b, 4d, 4e	2
	STATE 3	2024-10-28		4b, 4d, 4e	1
	STATE 2	2024-10-21		1/2b, 3b, 7	87
	STATE 5	2024-10-01		4b, 4d, 4e	1
	STATE 2	2024-09-26		1/2a, 3a	378
	STATE 2	2024-09-26		1/2a, 3a	91
	STATE 4	2024-09-25		1/2a, 3a	204
	STATE 1	2024-09-12		1/2c, 3c	9
	STATE 1	2024-09-12		1/2c, 3c	9
	STATE 4	2024-09-08		4b, 4d, 4e	1
	STATE 2	2024-09-08		1/2a, 3a	204
	STATE 4	2024-09-07		4b, 4d, 4e	1
	STATE 4	2024-09-03		1/2a, 3a	9

This report is intended solely for the named addressee, is confidential and may contain legally privileged information. The copying or distribution of this document or any information it contains, by anyone other than the addressee, is prohibited.

Table 3. All isolates of *Listeria* from NON-HUMAN sources received in the past 24 weeks for the reporting period ending 01/01/2025.

Sequence ID	State	Date Collected	NELSS ID	Molecular Serotype	MLST
	STATE 1	2025-01-30	Non-human	4b, 4d, 4e	1
	STATE 1	2025-01-30	Non-human	4b, 4d, 4e	1
	STATE 1	2025-01-23	Non-human	1/2b, 3b, 7	3
	STATE 1	2025-01-17	Non-human	1/2a, 3a	321
	STATE 1	2025-01-17	Non-human	1/2a, 3a	321
	STATE 1	2025-01-17	Non-human	1/2a, 3a	321
	STATE 1	2025-01-17	Non-human	1/2a, 3a	321
	STATE 1	2025-01-16	Non-human	1/2a, 3a	321
	STATE 1	2024-12-17	Non-human	4b, 4d, 4e	2
	STATE 1	2024-12-12	Non-human	4b, 4d, 4e	278
	STATE 1	2024-11-28	Non-human	4b, 4d, 4e	2
	STATE 1	2024-11-27	Non-human	1/2a, 3a	121
	STATE 1	2024-11-27	Non-human	1/2c, 3c	9
	STATE 1	2024-11-26	Non-human	1/2a, 3a	204
	STATE 1	2024-11-26	Non-human	1/2a, 3a	204
	STATE 1	2024-11-26	Non-human	1/2a, 3a	204
	STATE 1	2024-11-26	Non-human	1/2a, 3a	204
	STATE 1	2024-11-14	Non-human	4b, 4d, 4e	1
	STATE 2	2024-11-07	Non-human	1/2a, 3a	321
	STATE 2	2024-11-07	Non-human	1/2a, 3a	321
	STATE 2	2024-11-07	Non-human	1/2a, 3a	321
	STATE 2	2024-11-07	Non-human	1/2a, 3a	321
	STATE 2	2024-11-07	Non-human	1/2a, 3a	321
	STATE 2	2024-11-07	Non-human	1/2a, 3a	321
	STATE 1	2024-11-07	Non-human	1/2a, 3a	101
	STATE 1	2024-11-06	Non-human	1/2b, 3b, 7	3
	STATE 2	2024-11-03	Non-human	1/2a, 3a	321
	STATE 1	2024-10-16	Non-human	1/2b, 3b, 7	3
	STATE 2	2024-10-12	Non-human	1/2a, 3a	204
	STATE 2	2024-10-11	Non-human	1/2a, 3a	321
	STATE 2	2024-10-11	Non-human	1/2a, 3a	321
	STATE 2	2024-10-11	Non-human	1/2a, 3a	321
	STATE 2	2024-10-09	Non-human	1/2a, 3a	321
	STATE 2	2024-10-09	Non-human	1/2a, 3a	321
	STATE 2	2024-10-09	Non-human	1/2a, 3a	321
	STATE 3	2024-09-25	Non-human	4b, 4d, 4e	2
	STATE 1	2024-09-19	Non-human	1/2b, 3b, 7	5
	STATE 1	2024-09-17	Non-human	1/2c, 3c	9
	STATE 1	2024-09-13	Non-human	1/2a, 3a	204

This report is intended solely for the named addressee, is confidential and may contain legally privileged information. The copying or distribution of this document or any information it contains, by anyone other than the addressee, is prohibited.

Sequence ID	State	Date Collected	NELSS ID	Molecular Serotype	MLST
	STATE 1	2024-09-13	Non-human	1/2a, 3a	204
	STATE 1	2024-09-13	Non-human	1/2a, 3a	204
	STATE 2	2024-09-11	Non-human	4b, 4d, 4e	1
	STATE 1	2024-09-10	Non-human	1/2b, 3b, 7	3
	STATE 2	2024-09-06	Non-human	1/2a, 3a	204
	STATE 2	2024-09-06	Non-human	1/2a, 3a	14
	STATE 1	2024-09-05	Non-human	1/2a, 3a	321
	STATE 1	2024-09-05	Non-human	1/2a, 3a	321
	STATE 1	2024-09-03	Non-human	1/2a, 3a	204

Phylogenetic analysis is based on core genome single nucleotide polymorphisms (SNPs) and includes all isolates received in the past 12 months.

- Potential clusters are detected based on relationships observed in the phylogenetic tree.
- A recommendation is provided regarding these potential clusters.
- Epidemiological links should be investigated to support any possible clusters detected by SNP analysis

This report is intended solely for the named addressee, is confidential and may contain legally privileged information. The copying or distribution of this document or any information it contains, by anyone other than the addressee, is prohibited.

2. RESULTS

2.1 Epicurve

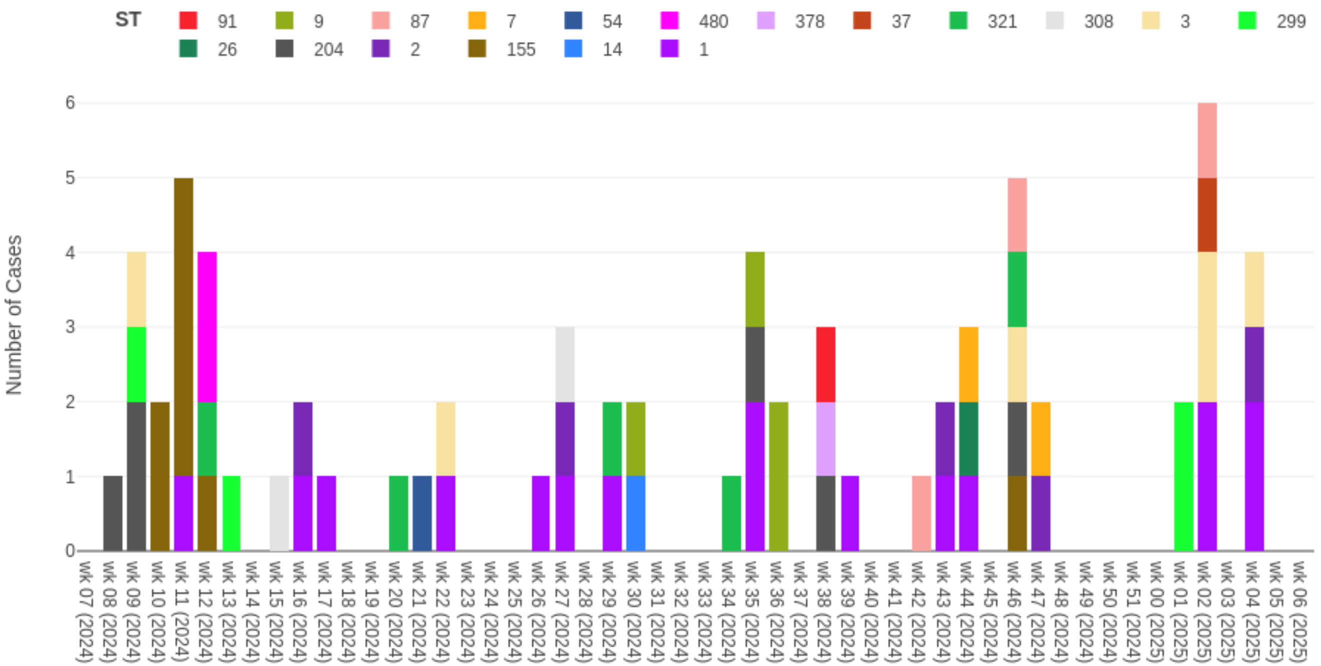

Figure 1. 12 month Listeriosis cases epicurve by sample collection date and multi-locus sequence type as determined from whole genome sequence data. Note, where sample collection date is not available sample submission date to NLRL is used. Isolates from non-sterile sample sites are not included.

This report is intended solely for the named addressee, is confidential and may contain legally privileged information. The copying or distribution of this document or any information it contains, by anyone other than the addressee, is prohibited.

2.2 Phylogenetic Cluster Analysis

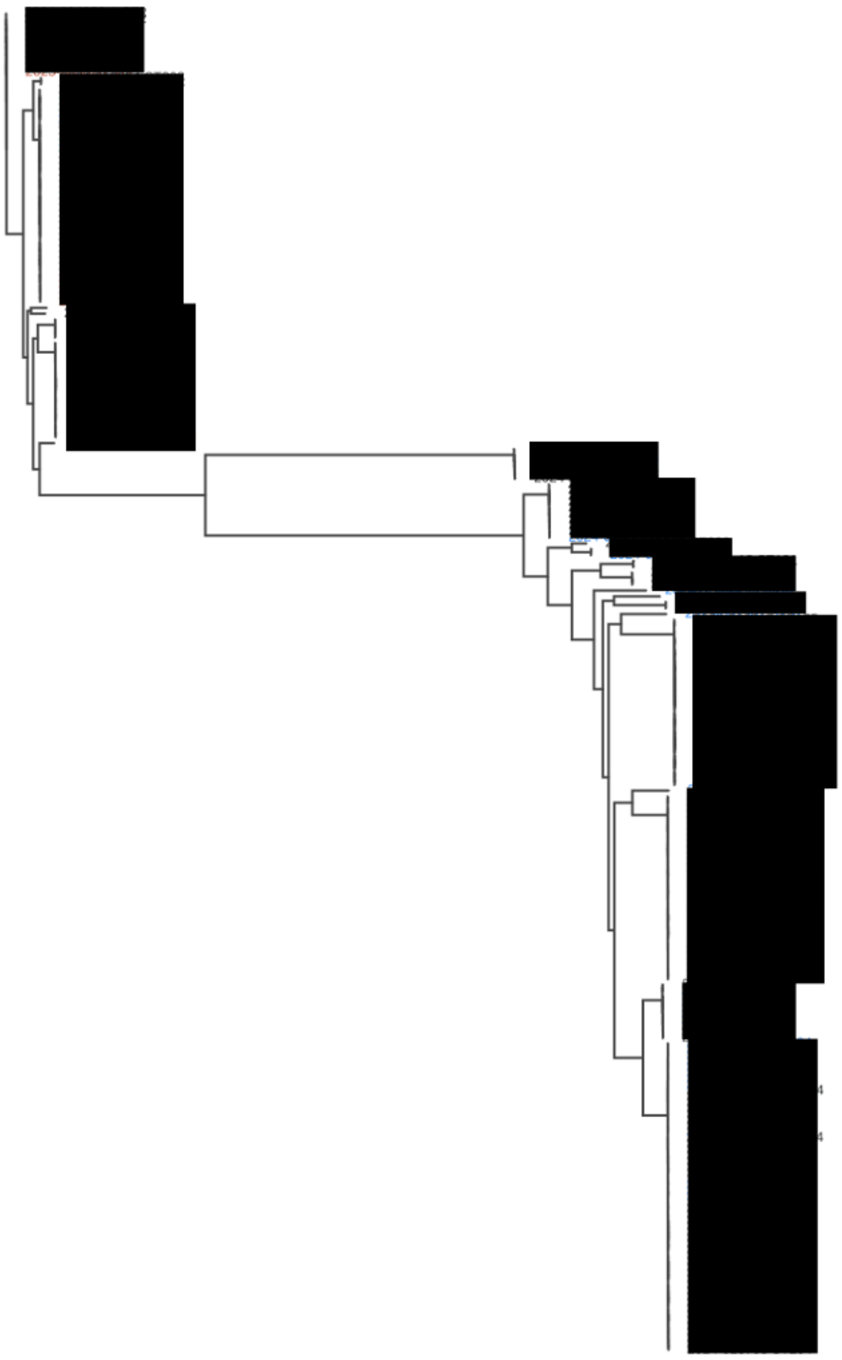

Figure 2. Phylogenetic analysis based on core genome SNPs of cases received in MDU PHL for reporting period ending 01/01/2025.

This report is intended solely for the named addressee, is confidential and may contain legally privileged information. The copying or distribution of this document or any information it contains, by anyone other than the addressee, is prohibited.

2.3 Re-analysis of potential clusters

Potential clusters identified in Figure 1 containing cases within the current reporting period are re-analysed with historical isolates of the same sequence types to determine whether any further investigation is required.

Cases are colour coded as follows:

**Red**	Recent cases are highly likely to be linked to other cases and further investigation is recommended to confirm these links.<br>Highly related clusters consist of isolates that were collected during the reporting period are linked at ≤ 5 SNP threshold.
**Orange**	Recent cases that are potentially linked to one or more cases and further investigation is recommended to confirm or reject the cluster.<br>Potentially related clusters consist of isolates that that were collected during the reporting period are linked at ≤ 20 SNP threshold.
**Green**	Recent cases that are not linked to any other cases therefore no further investigation is recommended.<br>Unrelated isolates do not cluster with other isolates that were collected during the reporting period at a threshold >20 SNPs
**Blue**	Cases identified with the last 12 months.
**Black**	Historical cases.

This report is intended solely for the named addressee, is confidential and may contain legally privileged information. The copying or distribution of this document or any information it contains, by anyone other than the addressee, is prohibited.

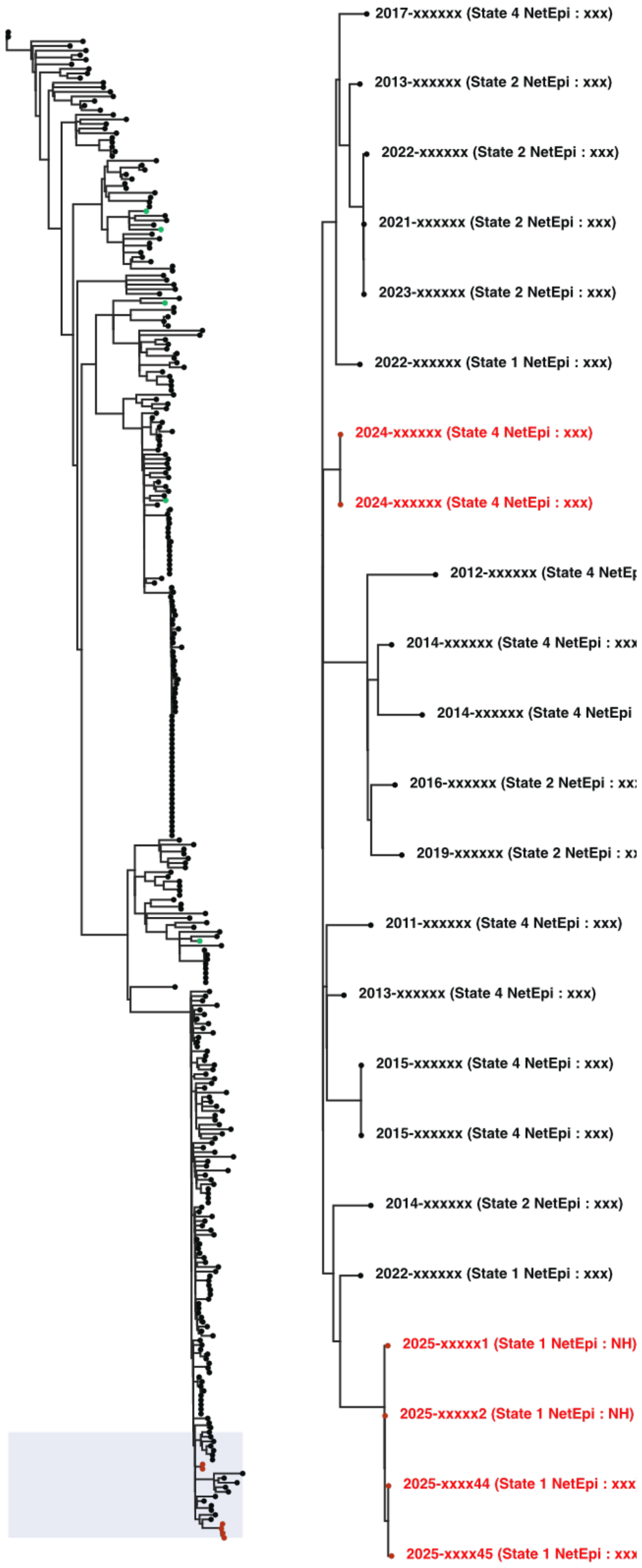

Figure 3. Phylogenetic re-analysis of **ST1** as identified in the initial full analysis – Full tree shown on left, highlighted region zoomed on the right.

This report is intended solely for the named addressee, is confidential and may contain legally privileged information. The copying or distribution of this document or any information it contains, by anyone other than the addressee, is prohibited.

3. METHODS

3.1 Procedure No(s)

Procedure No.	Service
SOP103	Whole genome sequencing
SOP109	Multilocus sequence typing (MLST)
SOP126	*In silico* analysis and reporting guidelines for *Listeria monocytogenes*
SOP147	SNP detection and interpretation of genomic relatedness

3.2 Determination of pair-wise SNP distances and cluster analysis

Phylogenetic trees were generated using a maximum-likelihood method, based on core genome alignment of all isolates analysed in this report. Pairwise SNP distances were also calculated from this alignment. The core genome alignments and clustering presented in this report are specific to this analysis and may change in subsequent analyses depending on the choice of reference, methodological approach employed and isolates included. Potential clusters were detected based on pairwise SNP distance thresholds current for *Listeria monocytogenes* analyses only. Criteria for relatedness is as follows:

- Highly related clusters consist of isolates that were collected during the reporting period are linked at ≤ 5 SNP threshold.
- Potentially related clusters consist of isolates that that were collected during the reporting period are linked at >5 and ≤ 20 SNP threshold.
- Unrelated isolates do not cluster with other isolates that were collected during the reporting period at a threshold >20 SNPs.

Maximum likelihood phylogenies, generated from core alignments, were annotated with clusters determined as described above. Recommendations regarding the degree of genomic relatedness are based only on the SNP distance matrix using a single-linkage analysis.

The following genomic references were used in the analyses presented in this report:

Sequence ID	Description
AE017262.2	Genome used as reference for Full phylogeny
AE017262.2	Genome used as reference for ST1
CP013288.1	Genome used as reference for ST2

This report is intended solely for the named addressee, is confidential and may contain legally privileged information. The copying or distribution of this document or any information it contains, by anyone other than the addressee, is prohibited.

Sequence ID	Description
CP006594.1	Genome used as reference for ST3
NZ_CP011004.1	Genome used as reference for ST4
CP014250.1	Genome used as reference for ST5
CP007685.1	Genome used as reference for ST7
CP013724.1	Genome used as reference for ST8
CP002003.1	Genome used as reference for ST9
CP001604.1	Genome used as reference for ST120
HG813249.1	Genome used as reference for ST121
AUSMDU00000235	Genome used as reference for ST14
AUSMDU00007774	Genome used as reference for ST155
CP011345.1	Genome used as reference for ST204
AUSMDU00012505	Genome used as reference for ST240
AUSMDU00007670	Genome used as reference for ST321
AUSMDU00012517	Genome used as reference for ST323

3.3 Bioinformatic Tools

The following bioinformatics tools were used in the analysis:

Tool	Version
Bohra	1.2.12
Snippy	4.4.5

This report is intended solely for the named addressee, is confidential and may contain legally privileged information. The copying or distribution of this document or any information it contains, by anyone other than the addressee, is prohibited.
