# Supplementary material for: Effects of Decentralized Sequencing on National Listeria monocytogenes Genomic Surveillance, Australia, 2016–2023
Source: Emerg Infect Dis. 2025 May;31(Suppl 1):S89–97. doi: 10.3201/eid3113.241357 (PMC12078541; doi:10.3201/eid3113.241357)
Supplement: Appendix 2 — Additional information on effects of decentralized sequencing on national Listeria monocytogenes genomic surveillance, Australia, 2016–2023. [file 24-1357-Techapp-s2.pdf]

Article DOI: https://doi.org/10.3201/eid3113.241357

EID cannot ensure accessibility for supplementary materials supplied by authors. Readers who have difficulty accessing supplementary content should contact the authors for assistance.

Effects of Decentralized Sequencing on National *Listeria monocytogenes* Genomic Surveillance, Australia, 2016–2023

Appendix 2

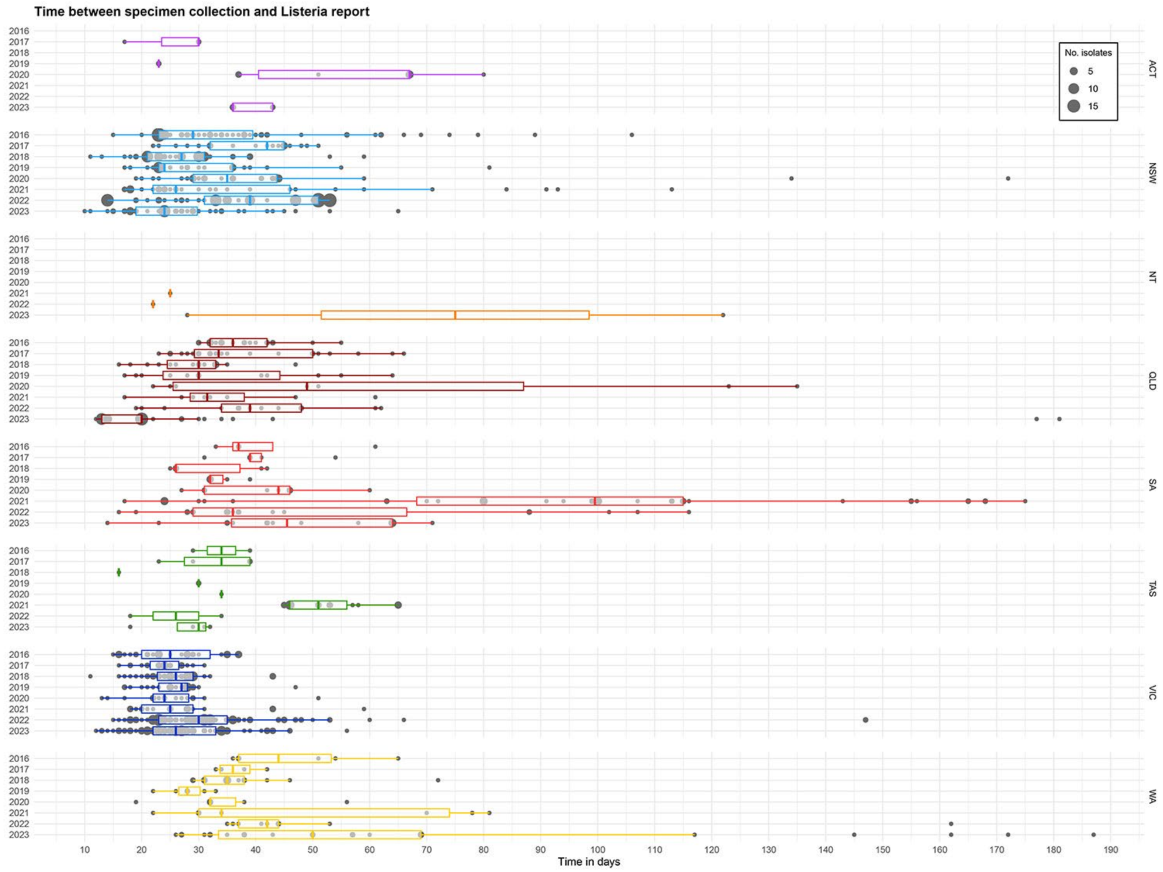

Appendix 2 Figure 1. System end-to-end timeliness – date of collection to date genomic surveillance report is issued by jurisdiction and year. Boxplots show median, 1st and 3rd quartiles, and whiskers indicate the values at 1.5*IQR from each quartile, with points outside this considered outliers. Underlying data are shown as dots, with the size corresponding to the number of samples at each time point. Boxplots are colored by jurisdiction, purple = Australian Capital Territory (ACT), skyblue = New South Wales (NSW), orange = Norther Territory (NT), maroon = Queensland (QLD), red = South Australia (SA), green = Tasmania (TAS), darkblue = Victoria (VIC), yellow = Western Australia (WA).

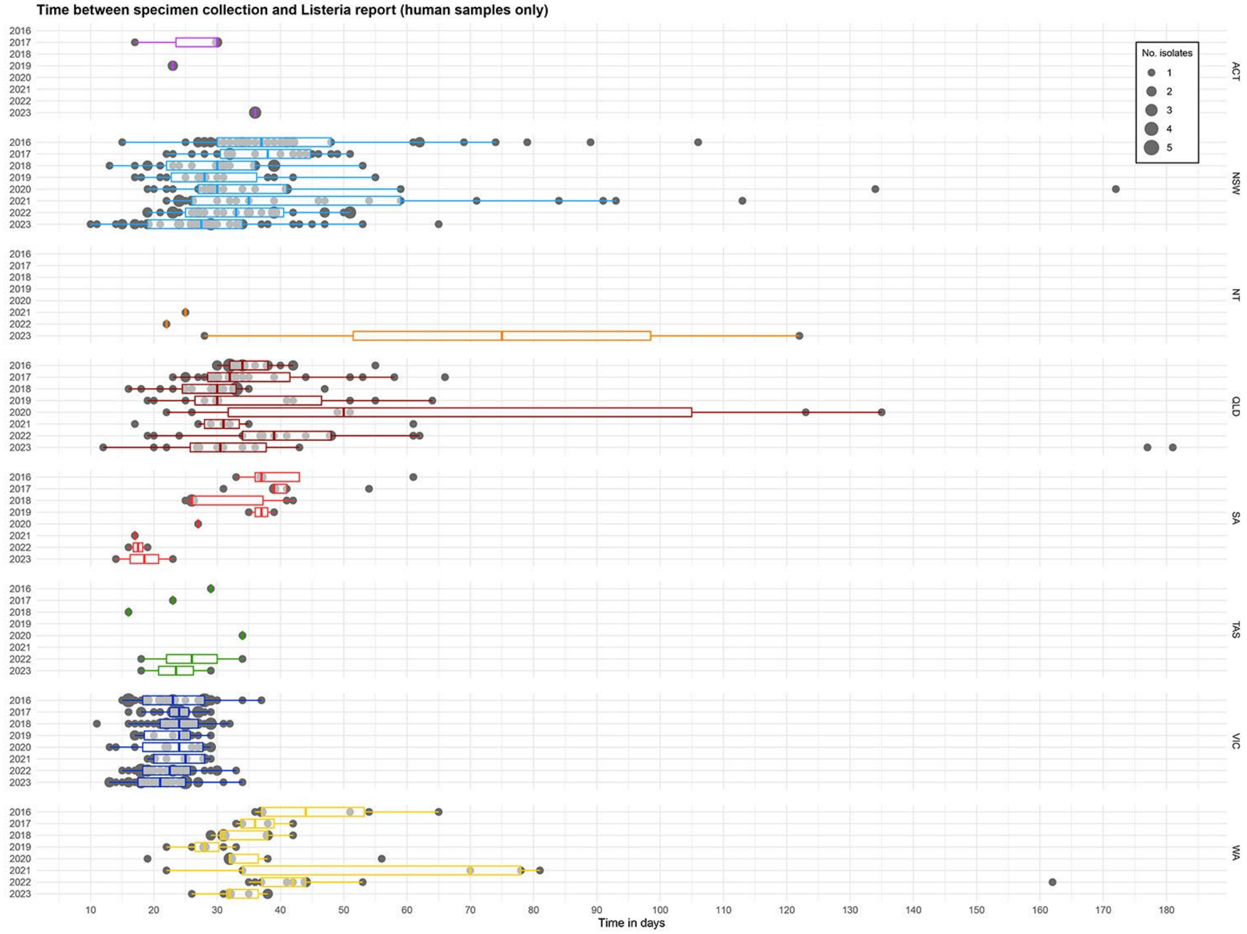

Appendix 2 Figure 2. System end-to-end timeliness – date of collection to date genomic surveillance report is issued for human samples only by jurisdiction and year. Boxplots show median, 1st and 3rd quartiles, and whiskers indicate the values at 1.5*IQR from each quartile, with points outside this considered outliers. Underlying data are shown as dots, with the size corresponding to the number of samples at each time point. Boxplots are colored by jurisdiction, purple = Australian Capital Territory (ACT), skyblue = New South Wales (NSW), orange = Norther Territory (NT), maroon = Queensland (QLD), red = South Australia (SA), green = Tasmania (TAS), darkblue = Victoria (VIC), yellow = Western Australia (WA).

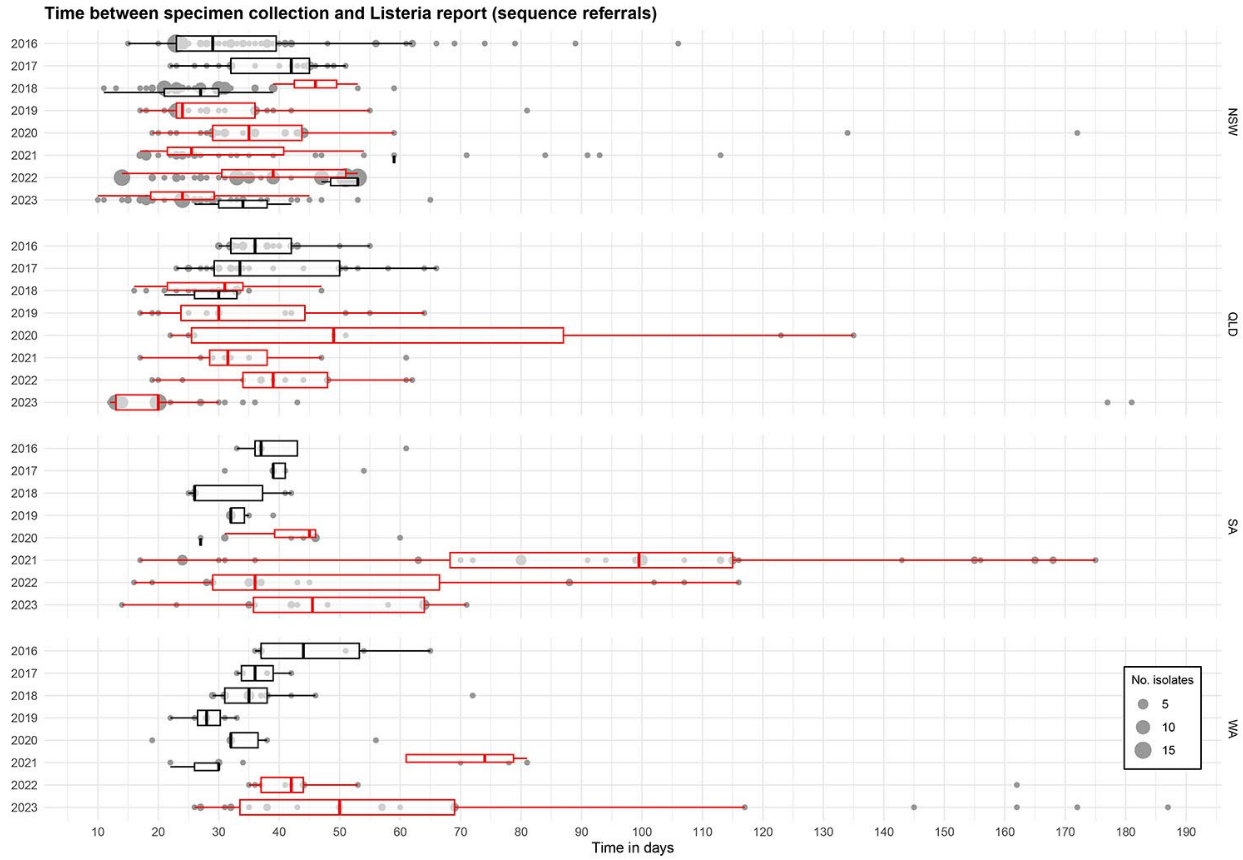

Appendix 2 Figure 3. System end-to-end timeliness – date of collection to date genomic surveillance report is issued for jurisdictions that had undergone transition to jurisdictional sequencing and referral of sequences, by jurisdiction, year and location of sequencing. Boxplots show median, 1st and 3rd quartiles, and whiskers indicate the values at 1.5*IQR from each quartile, with points outside this considered outliers. Underlying data are shown as dots, with the size corresponding to the number of samples at each time point. Boxplots are colored by jurisdiction, purple = Australian Capital Territory (ACT), skyblue = New South Wales (NSW), orange = Norther Territory (NT), maroon = Queensland (QLD), red = South Australia (SA), green = Tasmania (TAS), darkblue = Victoria (VIC), yellow = Western Australia (WA).

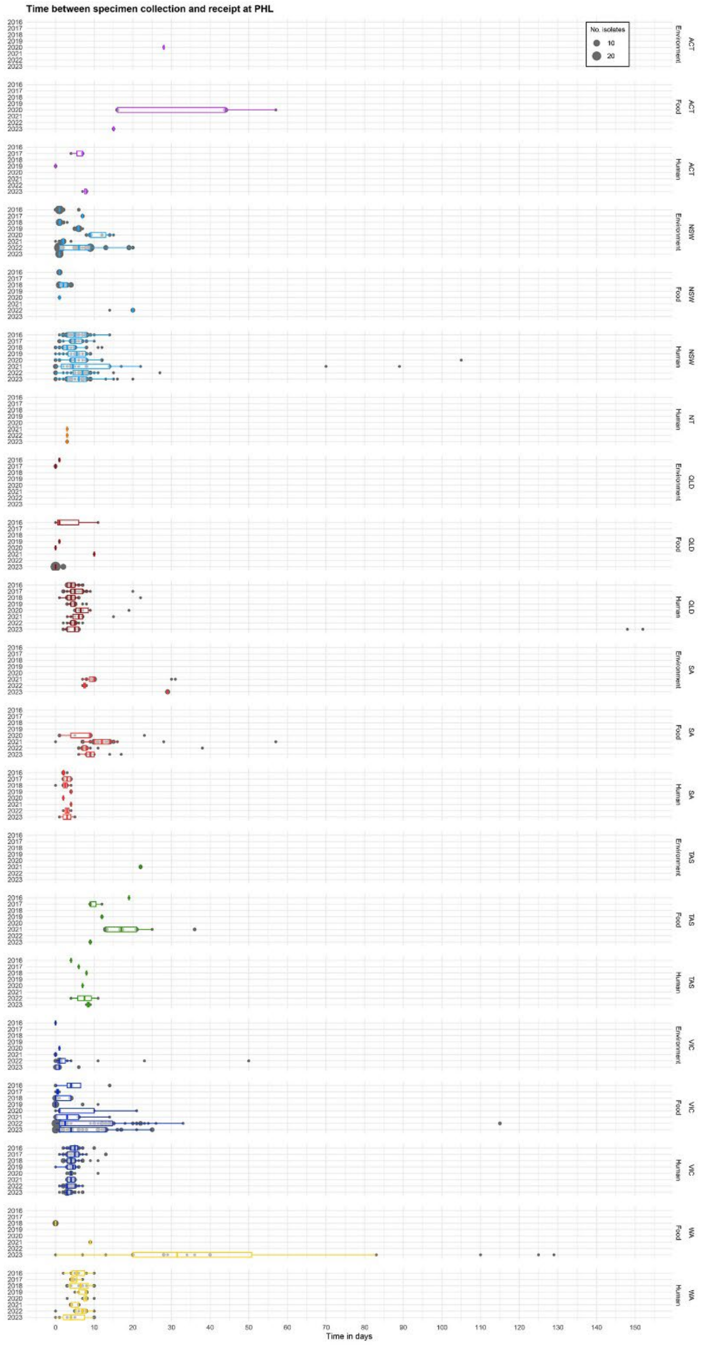

Appendix 2 Figure 4. Primary sample referral time – date of collection to date received at jurisdictional public health laboratory, by jurisdiction, sample source and year. Boxplots show median, 1st and 3rd quartiles, and whiskers indicate the values at 1.5*IQR from each quartile, with points outside this considered outliers. Underlying data are shown as dots, with the size corresponding to the number of samples at each time point. Boxplots are colored by jurisdiction, purple = Australian Capital Territory (ACT), skyblue = New South Wales (NSW), orange = Norther Territory (NT), maroon = Queensland (QLD), red = South Australia (SA), green = Tasmania (TAS), darkblue = Victoria (VIC), yellow = Western Australia (WA).

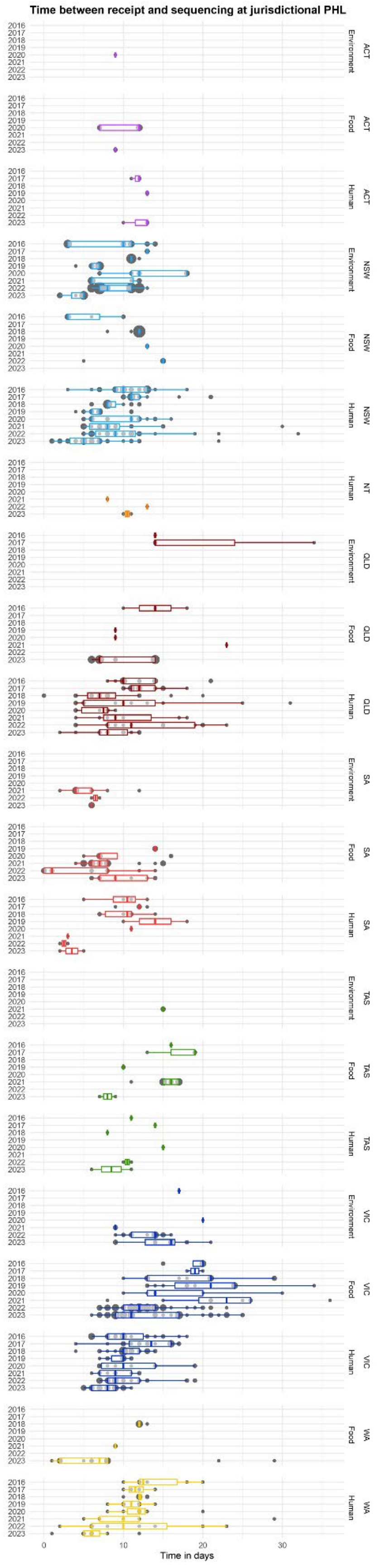

Appendix 2 Figure 5. Sequencing time – date received at jurisdictional public health laboratory to date sequenced, by jurisdiction, sample source and year. For samples from ACT, NT, TAS, VIC or samples pre-transition in NSW, QLD, SA and WA, the time is date received at NLRL to sequencing at NLRL. Boxplots show median, 1st and 3rd quartiles, and whiskers indicate the values at 1.5*IQR from each quartile, with points outside this considered outliers. Underlying data are shown as dots, with the size corresponding to the number of samples at each time point. Boxplots are colored by jurisdiction, purple = Australian Capital Territory (ACT), skyblue = New South Wales (NSW), orange = Norther Territory (NT), maroon = Queensland (QLD), red = South Australia (SA), green = Tasmania (TAS), darkblue = Victoria (VIC), yellow = Western Australia (WA).

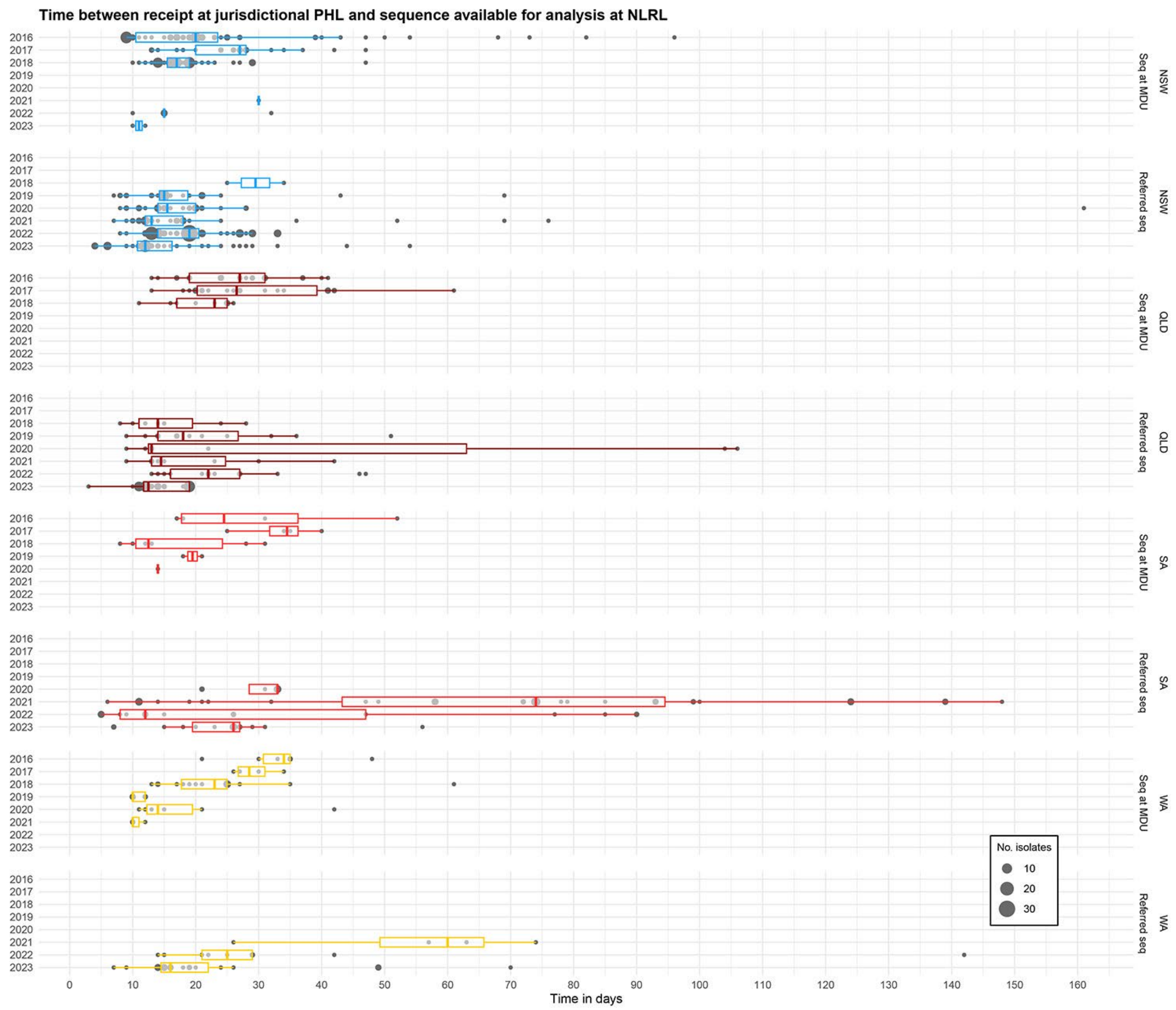

Appendix 2 Figure 6. Processing time for referred sequences – date received at jurisdictional public health laboratory to date sequence is available for bioinformatic analysis, accounting for processing times including potential culturing and isolation, and sequencing) either at the jurisdictional PHL or the NLRL, by jurisdiction, year and location of sequencing. Boxplots show median, 1st and 3rd quartiles, and whiskers indicate the values at 1.5*IQR from each quartile, with points outside this considered outliers. Underlying data are shown as dots, with the size corresponding to the number of samples at each time point. Boxplots are colored by jurisdiction, purple = Australian Capital Territory (ACT), skyblue = New South Wales (NSW), orange = Norther Territory (NT), maroon = Queensland (QLD), red = South Australia (SA), green = Tasmania (TAS), darkblue = Victoria (VIC), yellow = Western Australia (WA).

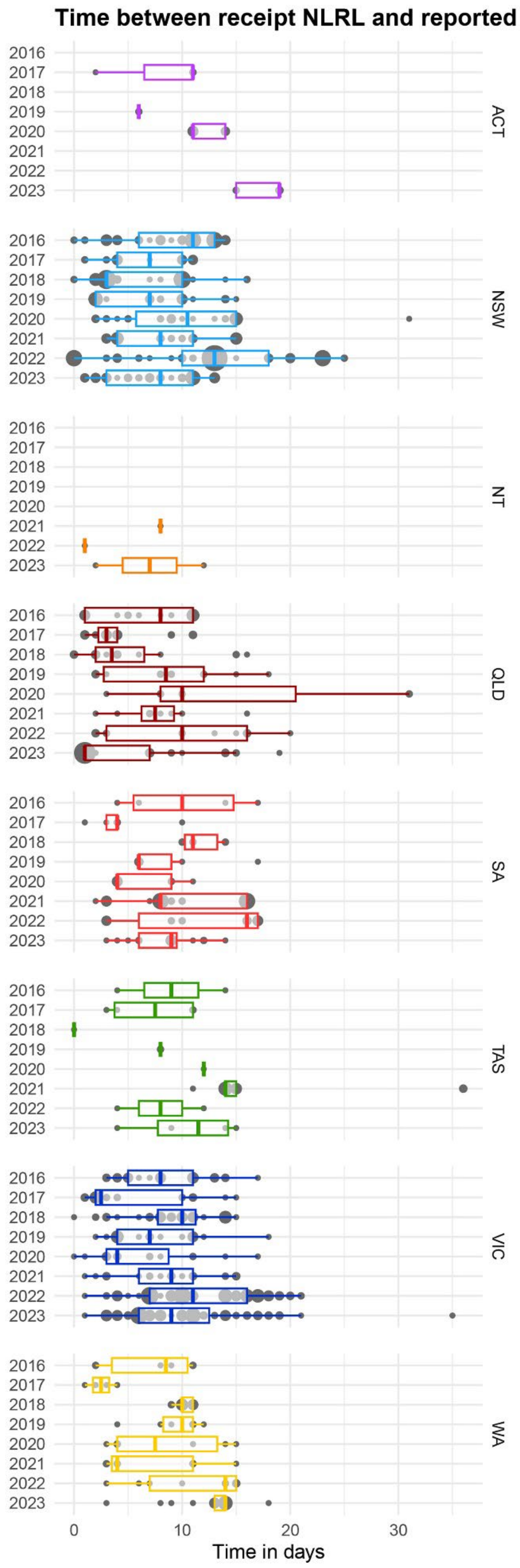

Appendix 2 Figure 7. Genomic analysis time – date a sequence was available (either completed sequencing at NLRL or sequence received at NLRL) and the date the fortnightly genomic surveillance report was issued, by jurisdiction and year. Boxplots show median, 1st and 3rd quartiles, and whiskers indicate the values at 1.5*IQR from each quartile, with points outside this considered outliers. Underlying data are shown as dots, with the size corresponding to the number of samples at each time point. Boxplots are colored by jurisdiction, purple = Australian Capital Territory (ACT), skyblue = New South Wales (NSW), orange = Norther Territory (NT), maroon = Queensland (QLD), red = South Australia (SA), green = Tasmania (TAS), darkblue = Victoria (VIC), yellow = Western Australia (WA).
